# Locus-Coeruleus Norepinephrine Functioning as a Predictor of Childhood Mental Health (LOCUS-MENTAL): Protocol for a Longitudinal Study

**DOI:** 10.2196/89237

**Published:** 2026-04-20

**Authors:** Iskra Todorova, Paula R Neubauer, Nico Bast

**Affiliations:** 1 Department of Child and Adolescent Psychiatry, Psychosomatics and Psychotherapy, Goethe-University, University Hospital Frankfurt, Deutschordenstraße 50, Frankfurt am Main, Hessen, 60528, Germany, 49 696301 ext 5279

**Keywords:** biomarker, pupillometry, transdiagnostic psychopathology, child development, locus coeruleus

## Abstract

**Background:**

Mental health disorders (MHDs) remain a leading cause of the global burden of diseases. Early identification of neurobiological mechanisms mediating a risk for MHDs is key to reducing a lifetime burden. Recent findings emphasize the locus coeruleus-norepinephrine (LC-NE) system as a neuromodulator of arousal translating acute stress responses into neuronal excitability. We propose that individual differences in LC-NE functioning can explain a differential susceptibility to psychological adversity, which mediates the development of transdiagnostic psychopathology in early childhood.

**Objective:**

The primary objective of LOCUS-MENTAL is to assess LC-NE functioning in preschoolers as a predictor of later psychopathology. This will be applied to generate an objective tool of individual early risk prediction, supporting targeted prevention of MHD.

**Methods:**

LOCUS-MENTAL includes 4 work packages. The centerpiece is an accelerated longitudinal study, in which a cohort of 300 preschool-aged children (aged 4‐6 years) will be recruited and followed up across 3 assessment waves, each one year apart. This will characterize developmental trajectories from 4 to 8 years of age. The primary outcome is the prediction of transdiagnostic psychopathology by pupillometry-derived LC-NE functioning. Transdiagnostic psychopathology is assessed by the Child Behavior Checklist (CBCL), while LC-NE functioning is assessed with pupillometry that includes core metrics of baseline pupil size (BPS) and stimulus-evoked pupillary response (SEPR). Cross-lagged panel models will be applied to the longitudinal data for quantifying causal effects between LC-NE functioning, childhood adversity, and psychopathology. Normative modeling and classification approaches will estimate an individual risk prediction based on pupillometric metrics of LC-NE functioning. The pupillometric battery has 4 passive auditory and visual paradigms. This battery will be validated with a multitrait-multimethod design that combines pupillometry with neurophysiological measures in electroencephalography, behavioral and cognitive measures, and neurocognitive paradigms. The study was approved by the Ethics Committee of the Faculty of Medicine at Goethe University Hospital (2024‐2160). Written informed consent will be obtained from caregivers and verbal assent by the children.

**Results:**

The study was funded in September 2024. Participant enrollment for the validation phase commenced in August 2025. As of February 2026, 82 participants were assessed, with a target of reaching 90 by the end of February 2026. Statistical analysis of the validation phase is planned for March 2026, with results aimed for publication in a peer-reviewed journal by the end of 2026. The longitudinal study is scheduled to start in April 2026 with completion in March 2029.

**Conclusions:**

The LOCUS-MENTAL study will establish whether LC-NE functioning provides a validated biomarker for detecting early psychopathology. The associated pupillometry provides a feasible and scalable test for identifying high-risk developmental trajectories in preschool children, which can be translated to clinical practice. The resulting risk assessment tool could facilitate a shift toward objective screening for mental health risks that enables targeted prevention with evidence-based treatment before diagnosis onset. This could prevent sequential comorbidity and reduce the lifetime burden of MHDs.

## Introduction

### Background

**Figure 1.**
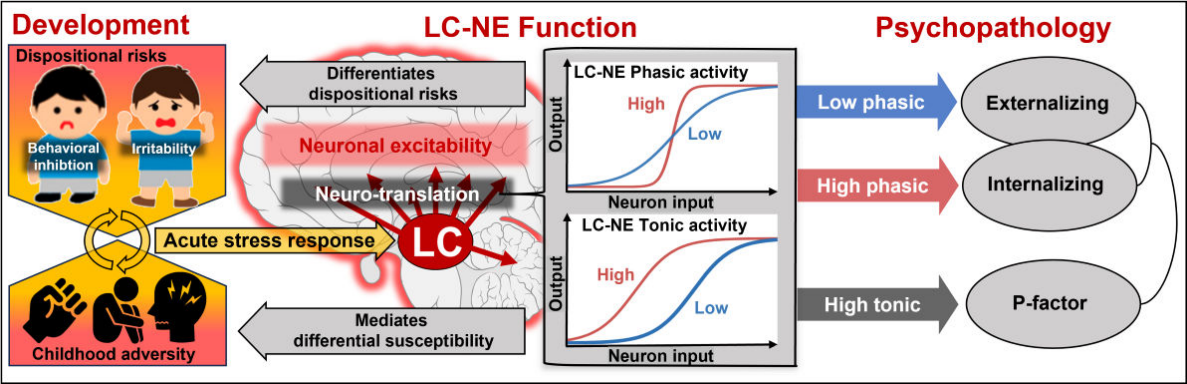
Theoretical model of LOCUS-MENTAL. Locus coeruleus-norepinephrine (LC-NE) functioning as a neurobiological mechanism in a developmental model of dispositional risks and stress, in which LC-NE functioning predicts psychopathology. LC-NE: locus coeruleus-norepinephrine.

Mental health disorders (MHDs) describe enduring altered cognition, emotion, and behavior and remain a leading cause of the global burden of diseases. Recently, the locus coeruleus norepinephrine system (LC-NE) has been established as a modulator of sensory processing that translates acute stress responses into neuronal excitability. We propose this LC-NE functioning to explain a differential susceptibility to psychosocial adversity that underlies dispositional risks for MHD (Figure 1). LOCUS-MENTAL will put this to the test. We will assess LC-NE functioning in preschoolers as a predictor of later psychopathology. We will use pupillometry as a feasible neuroimaging technique to quantify LC-NE functioning in preschool children. LOCUS-MENTAL ultimately aims to generate an objective tool of individual risk prediction that informs a targeted prevention of lifetime MHD.

LOCUS-MENTAL uses a transdiagnostic, dimensional, and developmental approach to investigate LC-NE functioning as a predictor of MHD. Variations of LC-NE functioning have been associated with phenotypes of psychopathology [1-3]. Our work validated pupillometry as a feasible method to assess LC-NE functioning, which will now be scaled to the application in an outpatient clinic for early detection in the general population [4,5]. Preschool children will be assessed in an accelerated longitudinal study to reveal prospective effects of LC-NE functioning on psychopathology in the school age. These key concepts are discussed below.

### Advancing Mental Disorders to a Transdiagnostic, Developmental Psychopathology

Comorbidity describes the co-occurrence of more than one diagnosis of MHD. Comorbidity is common, associated with more impairment and worse lifetime outcomes [6]. We lack the evidence to effectively treat MHD comorbidity at the time of this writing [7]. This may originate from conceiving MHD as independent diagnoses in clinical classification systems, which emphasized a focus on isolated diagnoses. In contrast, transdiagnostic psychopathology uses MHD comorbidity patterns to derive overarching factors. A bi-factor model with 3 factors showed the best fit in children [8]. An internalizing factor describes problems within individuals (depression and anxiety), whereas an externalizing factor describes problems between individuals (conduct problems and attention deficit hyperactivity disorder). Positive correlations between internalizing and externalizing established a superordinate p-factor that describes a liability for MHD. These 3 factors are continuous dimensions, in which manifestations below clinical thresholds are of prognostic relevance [9]. This transdiagnostic and dimensional perspective is expected to expose etiological mechanisms across MHD [10].

Comorbidity of MHD may further represent an endpoint where a secondary diagnosis arises because of a preceding primary diagnosis. This sequential comorbidity emphasizes a developmental perspective on MHD [11]. Most adults presenting comorbidity received an initial diagnosis in childhood, and half of all initial diagnoses occur before adolescence [6]. The preschool age may provide a sensible phase to identify mechanisms that influence developmental trajectories of lifetime psychopathology [12]. In addition, preschoolers differ in their risk for psychopathology even for shared psychosocial adversity [13]. This has been attributed to a differential susceptibility that explains an interaction of dispositional risks and stress [14]. We aim to identify a neurobiological mechanism of differential susceptibility [15] that mediates the impact of stress and contributes to the development of psychopathology.

### LC-NE in the Translation of Sensory Input to Neurophysiological Excitability

The LC-NE system is a neuromodulatory network orchestrating the brain’s response to sensory stimuli [16]. The LC-NE comprises the bilateral pontine locus coeruleus (LC) and its projections, which is the primary source of cerebral norepinephrine. Neuroimaging has suggested a bidirectional connectivity of the LC with the anterior cingulate cortex (ACC), thalamus, cerebellum, and the temporoparietal junction [17]. Compared to the substantia nigra, another neuromodulatory nucleus, the LC has shown increased connectivity to the visual, parietal, and motor cortices and the cerebellum [18]. This suggests a relative focus of the LC in earlier stages of sensory processing that perpetuates cognition and behavior.

The Adaptive Gain Theory (AGT) describes LC-NE functioning in cognition and behavior as a performance optimizer with 2 interchanging modes [19]. An exploration mode describes LC-NE tonic activity with a variable low frequency (1‐5 Hz) that corresponds to arousal levels [20]. In contrast, an exploitation mode describes LC-NE phasic activity with transient frequency and amplitude bursts that enhance the sensory processing of task-relevant stimuli [21]. At the system level, LC-NE activity reflects alerting attention [22]. At the neuron level, this has been elaborated to a mechanism of sensory selectivity. LC-NE tonic and phasic activity release norepinephrine which causes an autoinhibition of spontaneous neuronal activity and an excitation of input-driven activity [23]. This specific mechanism of neuronal gain modulation emphasizes a sensory processing of salience [24]. In the glutamate-emphasizes-noradrenergic-effects model [25], a salience is neurochemically indicated by a release of glutamate and can be independent of top-down control [24]. Under relative arousal, LC-NE activity initiates a positive feedback loop of glutamate and norepinephrine interactions that leads to excitatory hot spots of functional activity and biases large-scale networks [26] toward sensory processing of salient stimuli [27]. It has been observed that, in mice, a moderate stimulation of LC-NE activity enhanced thalamic feature selectivity, increased information transmission, and led to improved perceptual performance [28]. LC-NE functioning has been established as a key modulator of arousal and sensory processing with subsequent effects on perception and behavior [16,29].

### Pupillometry Reliably Measures LC-NE Functioning in Clinical Settings

LC-NE activity causes a dilation of the eye’s pupil. This pathway is indirect, as the LC-NE inhibits the Edinger-Westphal nucleus that typically emphasizes pupil constriction [30]. Pupil dilation has historically been used as an indicator of arousal and cognitive load [31]. Recently, pupil dilation has been differentiated into a baseline pupil size (BPS) and a stimulus-evoked pupillary response (SEPR), which index changes in LC-NE tonic and phasic activity, respectively [32]. These indices are assessed by pupillometry in video-based eye tracking. A study of combined functional magnetic resonance imaging and pupillometry showed a correlation of the LC’s signal with pupil dilation during the resting state (BPS) and with pupil dilation to oddball stimuli (SEPR) [33]. In a nonhuman primate model, an LC-NE stimulation predicted pupil dilation after 200‐300 milliseconds [34]. Other subcortical regions elicited pupil dilation with larger delays, which indicated an indirect activation of the LC [35]. In an optogenetic mouse model, pupil dilation marked relative changes in LC-NE activity, whereas variability challenges pupil size as a readout of absolute LC-NE activity [36]. This can be addressed by the application of baseline-corrected metrics [37]. In humans, the assessment of LC-NE activity is difficult with magnetic resonance imaging and electroencephalography (EEG) due to the LC’s location and small size [38]. This is pronounced in children that often induce more movement artifacts. Pupillometry now provides an index of LC-NE tonic and phasic activity (BPS and SEPR) that can be reliably measured with eye tracking. This innovative neuroimaging allows us to assess LC-NE functioning even in preschoolers in a clinical setting [5].

### LC-NE Functioning in an Etiological Model of Dispositional Risks and Stress

Psychopathology often develops by an interaction of dispositional risks and stress [39]. Dispositional risks describe internal features that increase a latent burden. In children, stress often refers to adverse childhood experiences like neglect and further extends to psychosocial domains like peer victimization [40]. If sufficient stress occurs upon dispositional risks, a psychopathology may develop. The preschool age is sensitive to this interaction and describes a precursor phase of lifetime psychopathology [15]. In preschoolers, dispositional risk profiles are observed, which map to polygenic scores for MHD that develop much later [11]. We propose that the LC-NE functioning in preschoolers underlies dispositional risks and shapes risk trajectories across diagnoses.

#### Dispositional Risks for Psychopathology and the LC-NE

Temperament describes a child’s innate sensory processing of affective stimuli and can represent an early dispositional risk [41]. Negative emotionality is a temperament with a proneness to negative affect that relates to later psychopathology and neuroticism. Negative emotionality has been associated with an elevated neurophysiological reactivity to reward and punishment, which explained an association between MHD polygenic scores and the p-factor in children [42]. This elevated reactivity has been reflected in an increased activity of the amygdala and the salience network and decreased activity of the ACC [43]. The LC-NE connectivity with the amygdala and ACC [44] suggests an effect of LC-NE functioning on negative emotionality.

#### Irritability

Irritability is a temperament trait of negative emotionality that is described by anger after frustration and attenuated fear under threat [45]. Irritability in children is the most pressing problem reported by help-seeking families [46]. Increased irritability predicted later conduct problems [47] and has been associated with a genetic risk for attention deficit hyperactivity disorder [48]. In contrast, irritability also predicted later internalizing [49]. This multifinality has been differentiated by error-related negativity (ERN) in EEG. In preschoolers with high irritability, increased ERN predicted later internalizing, while decreased ERN predicted later externalizing [50]. The ERN indicates an automated error detection that is modulated by LC-NE phasic activity [51]. Attenuated LC-NE phasic activity further attenuates a limbic fear response, which might promote externalizing behavior in irritable children [52]. We propose LC-NE phasic activity as an irritability correlate that differentiates between later externalizing and internalizing.

#### Behavioral Inhibition

Behavioral inhibition is another temperament trait of negative emotionality that is described by wariness and avoidance. Behavioral inhibition in young children predicted later anxiety and internalizing psychopathology as adults [53]. Behavioral inhibition in children has also been associated with an increased ERN [54] and an increased neurophysiological reactivity to novelty [55]. It indicates elevated LC-NE phasic activity as an amplifier of the limbic fear response that emphasizes a general threat perception and promotes behavioral inhibition [56]. We will investigate patterns of LC-NE activity that correspond to the early dispositional risks of irritability and behavioral inhibition. We expect differentiating profiles of LC-NE functioning, where elevated LC-NE phasic activity predicts later internalizing and attenuated LC-NE phasic activity predicts later externalizing psychopathology.

### LC-NE Activity as a Neuro-Translator of Acute Stress Responses

Stress like adverse childhood experiences is the other important determinant of psychopathology. An acute stress response is an internal coping mechanism to stress triggered by a neuronal network including the amygdala [57]. First, an autonomic response increases sympathetic activation for a momentary fight-flight-or-freeze response. A hypothalamic-pituitary-adrenal (HPA) axis then releases glucocorticoids like cortisol for sustained physical readiness. This endocrine stress response initially supports stress coping but cumulatively upregulates the HPA axis, which ultimately wears the body [58]. It explains the negative impact of stress over time. However, the acute stress response is also translated to a neuronal excitability that shapes our immediate perception in the face of stress [59].

This immediate neuro-translation of stress is moderated by LC-NE functioning [60]. The LC-NE has been established to initiate the autonomic stress response [61]. In addition, the HPA axis expresses a corticotropin-releasing hormone that binds to LC receptors and upregulates LC-NE tonic activity early in the endocrine stress response [62]. The upregulation of LC-NE tonic activity attenuates LC-NE phasic activity, which increases stimulus reactivity at the expense of sensory selectivity [63]. It reflects hyper-arousal [64] that intensifies the impact of stress [13]. For example, a liability to social stress in rodents was explained by an LC-NE modulation [65]. In humans, anxiety and stress-related disorders have been associated with increased LC-NE tonic activity [53,66]. Excessive LC-NE tonic activity in response to stress-inducing adversity might be an etiological mechanism of anxiety symptoms [62,66] that warrants an empirical evaluation in longitudinal studies.

In addition, LC-NE phasic activity is involved in the stress response by an LC projection to the amygdala in a subliminal response to fear [67]. LC-NE phasic activity emphasizes a neurophysiological response to emotional stress [68]. This has been explained by a different distribution of adrenergic receptors between the prefrontal cortex and the amygdala, where LC-NE phasic activity causes an inhibition of executive control and an excitation of the limbic fear response [69]. It has been observed in mice, pharmacological blocking of LC-NE phasic activity reduced amygdala activity during fear conditioning [70]. In a seminal study with healthy humans, LC-NE phasic responsivity and functional coupling with the amygdala during a neurocognitive task predicted internalizing symptoms following stress-inducing life events [2]. Accordingly, posttraumatic stress disorder has been associated with elevated LC-NE phasic activity in response to loud noises [71]. This supports increased LC-NE phasic activity as an amplifier of the neurophysiological fear response in the face of stress.

LC-NE activity in stress regulation can, thus, be described as a (neuro-)translation of the acute stress response to neuronal excitability [72]. We propose that LC-NE functioning as a mechanism in young children that shapes a subjective perception of stress. Distinct LC-NE functioning profiles may emphasize psychological states (eg, threat perception) that increase the psychopathological impact of stress. Thus, interindividual differences in LC-NE functioning are expected to mediate the negative impact of childhood adversity. We expect that elevated LC-NE tonic activity corresponds to hyperarousal under stress that represents a liability to transdiagnostic psychopathology (p-factor). We further expect deviant LC-NE phasic activity to differentiate the development of internalizing versus externalizing psychopathology in response to childhood adversity. Elevated LC-NE tonic and phasic activity have been associated with internalizing symptoms in adults (eg, anxiety and negative affect), whereas blunted LC-NE phasic activity might indicate a subtype at risk for conduct problems [73]. LOCUS-MENTAL will investigate LC-NE functioning in preschoolers that mediates a differential susceptibility to adversity and differentiates future profiles of transdiagnostic psychopathology (see Figure 1).

### Preliminary Work

#### Validity of Pupillometry in Clinical Samples

**Figure 2.**
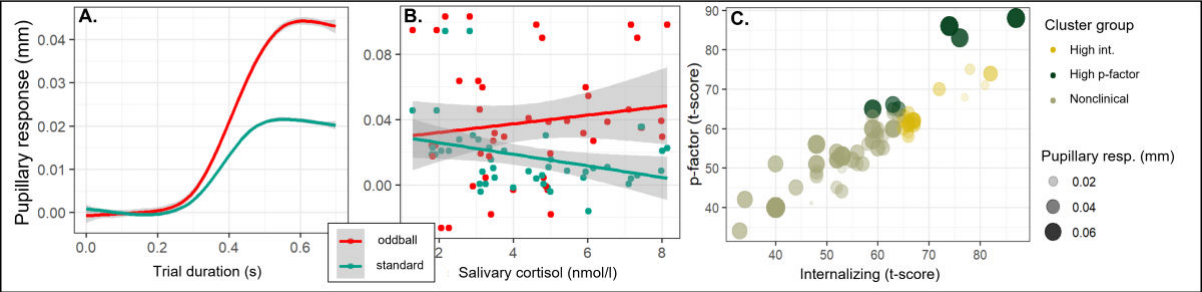
Preliminary work on auditory oddball task. (A) Stimulus-evoked pupillary response (SEPR) of children in an auditory oddball task. (B) SEPR to oddball stimuli as an index of LC-NE phasic activity is associated with salivary cortisol levels. (C) SEPR to oddball stimuli differentiated between factors of transdiagnostic psychopathology within clinically relevant ranges.

We established a model that introduced LC-NE activity as a modulator of attentional function in autism [74,75]. We manipulated task utility to induce changes in pupillary responses that related LC-NE functioning to performance [1]. We established the LC-NE to influence memory [76] and social attention [77]. At the time of this writing (DFG number: 492582254), we validate LC-NE measures by a combined pupillometry and EEG study. In data of 150 children, we showed a higher SEPR for oddball versus standard stimuli in a passive auditory oddball task (manuscript under review). In a recent publication, we used the European Autism Interventions Longitudinal European Autism Project sample [78] to analyze combined pupillometric and EEG data in a large cohort of deeply phenotyped autistic and nonautistic individuals. We showed that baseline BPS and SEPR as pupillometric measures drive the event-related potential of mismatch negativity in the response to oddball sounds [4]. This well-investigated auditory oddball task is applied as a reference task in this research program (Figure 2A).

#### Reliability of Pupillometry in Preschoolers

We showed the feasibility of our methodology in early childhood by acquiring longitudinal pupillometric data in an eye-tracking battery of neurocognitive tasks in 100+ preschool children. Two cross-sectional analyses have been published [79,80], while a longitudinal study applied pupil responses as a mediating mechanism in early intervention outcomes [5]. We assessed the reliability of pupillometry in 26 participants with 3 measurements (baseline, after 6 months, and after 12 months). BPS showed high correlations between measurement time points (*r*s=0.69−0.81) and an intraclass correlation of ICC=0.90, 95% CI 0.83-0.95. SEPR showed lower correlations between measurement time points that might be induced by intervention effects.

#### Pupillometry and Stress Response

We further assess pupillometry and stress responses during an oddball task in adolescents (32-01/22). In preliminary data (n=32), BPS was associated with an attenuated downregulation of salivary cortisol (*β*=.56, 95% CI 0.38-0.74; *R*^2^=0.28), while pupillary response to oddballs (SEPR) was associated with higher hair (Δ*β*=.24, 95% CI 0.07-0.42; *R*^2^=0.15) and salivary cortisol levels (Figure 2B) supporting the LC-NE in stress responses. Finally, SEPR differentiated psychopathology above clinical cutoffs (Figure 2C). Taken together, our preliminary work underlines the feasibility and reliability of pupillometry in preschoolers, outlines our capacity to recruit burdened preschoolers, and emphasizes pupillometric responses as an index of LC-NE activity in stress and psychopathology.

### Main Objectives

**Figure 3.**
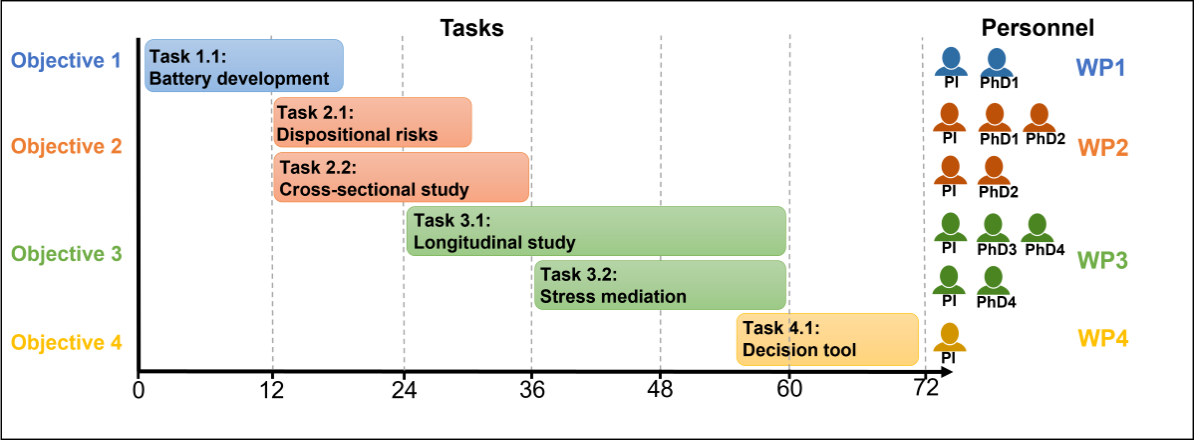
Work program of LOCUS-MENTAL with time flow, required personnel, and the associated tasks.

The primary aim of LOCUS-MENTAL is to establish pupillometric measures of LC-NE functioning as a predictor of transdiagnostic psychopathology in childhood. We hypothesize that LC-NE functioning represents a fundamental neurobiological mechanism in the development of MHD that could serve as a biomarker for early risk detection. Transdiagnostic psychopathology is assessed with the caregiver-reported Child Behavior Checklist (CBCL), where clinical relevance is defined as reaching established cutoffs (T>60). LC-NE functioning is assessed with pupillometry. The study outcome could improve targeted prevention and intervention for MHD comorbidity in children. To achieve this aim, LOCUS-MENTAL is structured into 4 consecutive objectives, each with specific tasks within the following project plan (Figure 3).

#### Objective 1

This study aims to validate a concise test battery assessing LC-NE system functioning in preschool children using pupillometric measures during passive tasks. The test battery will be evaluated for convergent and discriminant validity.

#### Objective 2

This study aims to associate LC-NE functioning with correlates of psychopathology in a cross-sectional study. Task 2.1 will determine whether LC-NE functioning profiles serve as markers of dispositional risks and differentiate between irritability and behavioral inhibition. Task 2.2 will examine whether LC-NE functioning further mediates the association between childhood adversity and transdiagnostic psychopathology.

#### Objective 3

This study aims to evaluate the prospective effect of LC-NE functioning in a developmental model of vulnerability and stress. Task 3.1 will investigate whether pupillometric measures of LC-NE functioning in preschool age predict risk for psychopathology at school age. Task 3.2 will assess LC-NE functioning as a mediator between early stress responses and later psychopathology.

#### Objective 4

This study aims to develop an objective tool of risk prediction in the preschool ages based on the pupillometric measures of LC-NE functioning. LC-NE function profiles in preschoolers define a normative development space, where individual deviations indicate quantitative risks for MHD.

## Methods

### Overview

**Figure 4.**
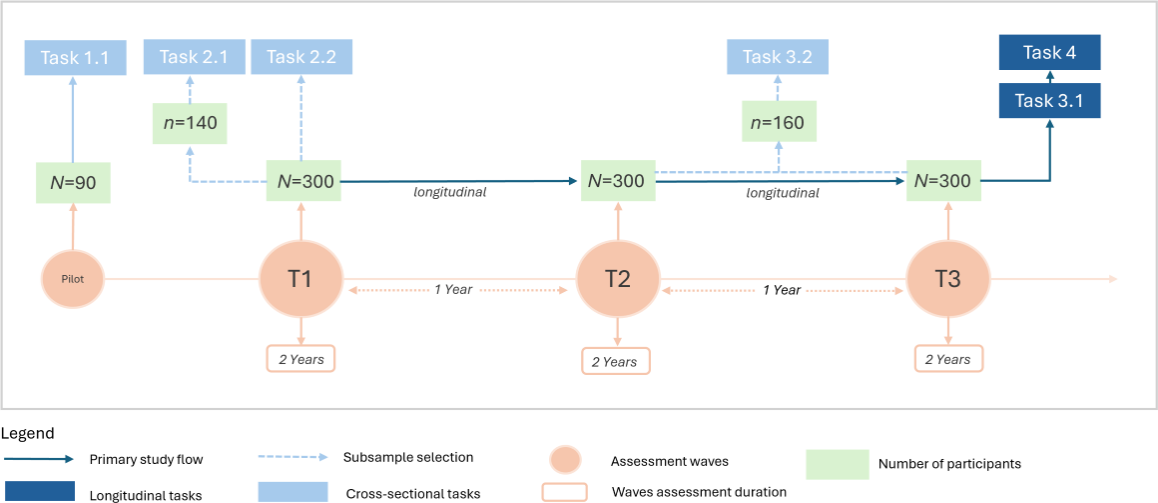
LOCUS-MENTAL study design with task integration. The flowchart illustrates the longitudinal study design consisting of a pilot phase and 3 primary assessment waves (**T1, T2, AND T3**) each one year apart. Each assessment wave spans over 2 years. The longitudinal study will follow a cohort of 300 preschool children (initially aged 4‐6 years). Dark blue boxes indicate longitudinal study elements, and light blue boxes indicate cross-sectional tasks within the longitudinal study. Dotted lines indicate selection of a specific subsample at specific time points, including a randomized subsample (n=160) either from T2 or T3 for Task 3.2. Small “n” represents the subsample number, while “N” represents the primary study sample.

LOCUS-MENTAL will achieve its objectives by combining pupillometric indices of LC-NE functioning and using a prospective panel study design (Figure 4). Pupillometry is assessed by video-based eye tracking during basic paradigms that are feasible in preschoolers. Transdiagnostic psychopathology is assessed by CBCL externalizing, internalizing, and total T-scores. Childhood adversity is assessed by interviewing caregivers, children, and clinicians. Pupillometric indices, transdiagnostic psychopathology, and childhood adversity are repeatedly measured in an accelerated longitudinal study: We assess preschoolers (aged 4‐6 years) in an initial assessment with reassessments after one year (aged 5‐7 years) and after 2 years (aged 6‐8 years). This accelerated design allows revealing causative effects of LC-NE functioning on psychopathology from 4 to 8 years of age. In addition, this allows quantifying LC-NE functioning as a mediator in the effect of childhood adversity on psychopathology. In distinct subtasks, LC-NE functioning is elaborated as a neurophysiological mechanism of established dispositional risks, while LC-NE functioning in stress regulation is explored by combined pupillometry and analysis of hair cortisol levels and salivary cortisol after a social stress test. This will characterize LC-NE functioning profiles in the interaction of dispositional risk and stress. Finally, LOCUS-MENTAL applies normative modeling on the pupillometric data to develop a tool for risk prediction of developmental trajectories in transdiagnostic psychopathology. An overview of all measures is provided below (Table 1). The reporting of this study protocol follows the STROBE (Strengthening the Reporting of Observational Studies in Epidemiology) guidelines for longitudinal cohort studies [81]. The completed checklist is available in Checklist 1.

**Table 1.** Assessment measures and questionnaires of LOCUS-MENTAL.

Modality, instrument and task		Construct	Objective
Questionnaires (caregiver)	
core set		Demographic data	1.1.‐3.2
CBCL^a^ [4-18,82]		Psychopathology	2.1‐3.2
BIQ^b^ [83]		Behavioral inhibition	2.1‐3.2
ARI^c^ [84]		Irritability	2.1‐3.2
CBQ-VSF^d^ [85]		Temperament	2.1‐3.2
ACE+^e^ [86]		Childhood Adversity	2.2‐3.2
F-SoZu 6^f^ [87]		Social support	2.2‐3.2
Interview (child)	
CLES-P^g^ [88]		Childhood adversity	2.2‐3.2
VEX-Preschool^h^ [89]		Violence exposure	2.2‐3.2
Pupillometry battery	
Auditory oddball [90]		LC-NE activity	1.1‐3.2
Visual oddball [91]		LC-NE activity	1.1‐3.2
Cued visual search [92]		LC-NE activity	1.1‐3.2
Rapid sound sequences [24]		LC-NE activity	1.1‐3.2
Cognitive tests	
WPPSI^i^ subtest–vocabulary [93]		Verbal IQ estimate	1.1‐3.2
WPPSI subtest–block design [93]		Nonverbal IQ estimate	1.1‐3.2
Bio samples	
Hair sample		Retrospective cortisol level	2.2‐3.2
Saliva sample		Acute cortisol level	2.2‐3.2
Assessment	
Child stress test [94]		Stress reaction	3.2
EEG^j^	
See pupillometry tasks		LC-NE activity	1.1‐2.1
Go/NoGo Task [50]		Error Related Negativity (ERN)	2.1
Neurocognitive tasks	
Dot-probe tasks [95]		Irritability	2.1
Frustrative reward learning task [96]		Irritability	2.1

a CBCL: Child Behavior Checklist

b BIQ: Behavioral Inhibition Questionnaire

c ARI: Affective Reactivity Index

d CBQ-VSF: Children’s Behavior Questionnaire–Very Short Form

e ACE+: Adverse Childhood Experiences Questionnaire Plus

f F-SoZu 6: Social Support Questionnaire

g CLES-P: Childhood Life Events Scale–Preschool

h VEX-Preschool: Violence Exposure Scale–Preschool

i WPPSI: Wechsler Preschool and Primary Scale of Intelligence

j EEG: electroencephalogram.

### Recruitment

The main recruitment avenues will be the outpatient clinic for early detection at the Department for Child and Adolescent Psychiatry at the Goethe University Hospital. In the help-seeking population, externalizing disorders might be overrepresented, which is why we will overrecruit preschoolers with internalizing symptoms. We aim for a representative sample by recruitment across all levels of psychopathology. This is why we will also recruit in the general population by distributing flyers with study information at local health care and public institutions, pediatricians, and day care facilities and promoting the study in targeted social media groups and channels. Exclusion criteria will be known genetic syndromes and neurological diseases. Caregivers will receive a summary report of their child’s assessment (eg, IQ evaluation) upon completion of each assessment wave.

### General Setting

The experimental assessments will be carried out at our combined eye-tracking and EEG lab that we established at the clinic. The procedure is framed as a play experience. The child watches a sequence of their favorite movie on the stimulus presentation screen. This is followed by the assessments where the child may sit on their caregiver’s lap. Each paradigm is designed to enable breaks. Questionnaire measures may be filled out by the caregivers via our in-house online tool to decrease the participant burden, whereas structured clinical interviews and child questionnaires will be carried out by trained clinical staff.

### Sample Size and Power

Sample sizes (n) were determined based on recruitment feasibility and the ability to detect moderate effects with a power of *β*>.80. For each task, we estimated the power by Monte Carlo simulations (k=1000) within respective models [97,98]. In the following sections, the sample size and power are reported, respectively, for each specific objective or task.

### Work Package 1 (Objective 1): Validation of a Pupillometric Battery of LC-NE Functioning

#### Participants

We will recruit 90 preschool-aged children to participate in a passive pupillometry test battery designed to measure LC-NE activity.

#### Sample Size and Power

A power analysis using a linear mixed model (LMM) indicates a power of *β*=.82 for 90 participants to detect a moderate stimulus effect (b=0.3) in the auditory oddball task (100 trials, 20% oddballs).

#### Experimental Design

This task uses a multitrait, multimethod design combining pupillometry, EEG, and behavioral assessments to validate the pupillometry battery.

#### Battery Design

##### Overview

The battery consists of 4 individual tasks. The primary task is a passive auditory oddball task, and the 3 additional tasks are a rapid sound sequence, a visual oddball, and an audio-cued visual search task. At the beginning of each task, a fixation cross in the center of the screen is presented for 5 seconds and can be used as a global baseline. After the last task, there is an additional measurement of a 5-second baseline. All tasks are presented in a fixed order, and in between the tasks, a short children’s cartoon (duration=30 s) is presented to maintain children’s attention and motivation. The overall duration of the battery is approximately 16 minutes. Prior to the task, we conduct a 6-point calibration. The source code of the tasks as a Python (Python Software Foundation) implementation can be found on the GitHub repository “locusmental_wp1_tasks” of Nico Bast.

##### Auditory Oddball Task

A passive auditory oddball task that is feasible in preschoolers (see 1.3, Figure 2) will be applied as a reference measure [90]. The auditory oddball task consists of a series (k=100) of frequent (“standard,” 80%) or infrequent (“oddball,” 20%) pure tones. Pure tones will have a short duration (50 ms) and will be presented with an interstimulus interval of a random duration between 1.8 and 2 seconds, while the pitch of the pure tones (500 or 750 Hz) will be counterbalanced between participants. Attention will be directed to the screen center by a continuous cartoon video without sound and controlled for by gaze-contingent trial presentation. Deviant gazes will evoke an attention-grabbing stimulus to redirect attention.

##### Rapid Sound Sequences Task

The Rapid Sound Sequences task consists of auditory stimuli 6 seconds-long sequences of subsequent tone pips (50 ms duration), drawn from a pregenerated pool of 20 fixed frequencies. Each sequence is followed by a 2-second interstimulus interval. The tone-pip sequences are arranged according to varying patterns and are randomly generated for each participant and on each trial. Conditions vary by levels of auditory regularity and irregularity with 2 control and 3 transition conditions. Previous research showed that transitions from regularity to irregularity evoked pupillary responses [24].

- Control condition regular 10 (REG10): randomly selecting 10 frequencies from the pool, arranging them in a fixed sequence pattern, and iterating the sequence to create a regular pattern.
- Control condition random 20 (RAND20): randomly selecting 20 frequencies with replacement from the pool and playing these fully randomized.
- Transition condition REG10-RAND20: the first 3 seconds are presented as REG10, followed by a transition to RAND20 for 3 seconds.
- Transition condition RAND20-REG10: the first 3 seconds are presented as RAND20, followed by a transition to REG10 for 3 seconds.
- The transition condition RAND20-REG1: the sequence starts as RAND20 for 3 seconds and transitions into REG1, a single tone pip selected from the pool and iterated for the final 3 seconds.

Each control condition will be presented 5 times, and each transition condition 10 times, in randomized order. Attention will be directed to the screen center by a fixcross or a video and controlled for by gaze-contingent trial presentation. Deviant gazes will evoke an attention-grabbing stimulus to redirect attention. A silent cartoon will be presented during the interstimulus interval.

##### Visual Oddball Task

The visual oddball task consists of 72 trials displaying frequent (standard size: 2.9° visual degrees; 80%) and infrequent (oddball size: 4.6° visual degrees; 20%) blue circles. Each stimulus is displayed for a 150-millisecond duration and is preceded by a 1.5-second interstimulus interval. Attention is maintained using gaze-contingent trial presentation.

##### Audio-Cued Visual Search Task

Each trial presents a visual search display consisting of 4 colored circles (red, yellow, or green) arranged in an imaginary circle around the center of the screen. One circle (target circle) has a deviant color from the 3 distractors, and its position changes randomly across the 30 trials. The visual search phase has a duration of 1.5 seconds. An auditory alerting cue, a simple brief beep, is presented in 50% of the trials. The cue duration varies randomly between 200 and 300 milliseconds and is presented with a random delay of 0‐100 milliseconds within a 400-millisecond interval prior to the visual search onset. Each trial starts with a 1.5-second interstimulus interval with a fixation cross in the center of the screen. Attention is maintained using gaze-contingent trial presentation.

### Neurophysiological Measures

BPS and SEPR are assessed for each trial in each task. BPS is defined as the pupil size prior to stimulus onset, while SEPR represents a pupil size change in response to stimuli. BPS and SEPR will be evaluated in the auditory oddball task in response to standard trials as neurophysiological habituation [99] and to oddball trials as sensory selectivity [33].

### Cognitive Measure

To assess the verbal and cognitive abilities, we will conduct 2 subtests from the Wechsler Preschool and Primary Scale of Intelligence (WPPSI) [93].

### Statistical Analysis Plan

**Figure 5.**
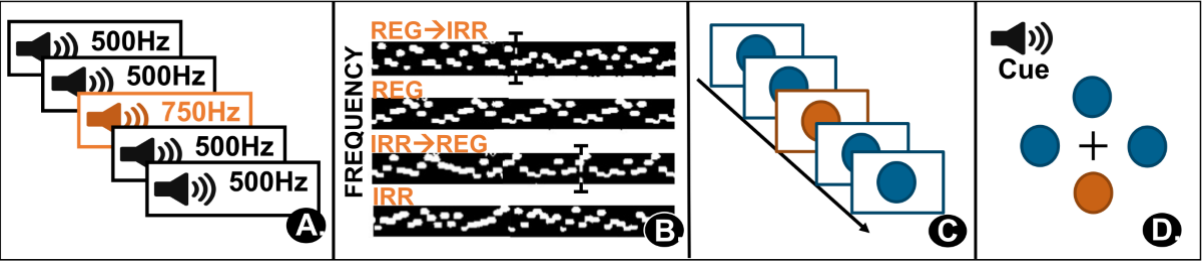
Experimental tasks. (**A**) Auditory oddball task (see description above). (**B**) Irregularity in rapid sound sequences; sequences either have a pattern (regular=REG), have no pattern (irregular=IRR), or change regularity within the sequence (REG to IRR, IRR to REG). (**C**) Visual oddball task. (**D**) Cued and uncued visual search task. IRR: irregular; REG: regular.

We will validate the pupillometric indices by a multitrait-multimethod design. In a homo-method analysis, the BPS and SEPR during the auditory oddball task (Figure 5A) will be compared to BPS and SEPR in tasks of LC-NE functioning with irregularity in rapid sound sequences [24] (Figure 5B), a visual oddball task [91] (Figure 5C), and an audio-cued visual search [92] (Figure 5D).

In a hetero-method analysis, the BPS and SEPR during the auditory oddball task will be compared to event-related potentials (ERPs) in concurrent EEG. We investigate oddball ERPs that are likely associated with LC-NE activity (P300, MMN) [27,100]. Finally, the pupillometric measures will be compared to divergent measures of cognitive and language ability as assessed by WPPSI-IV [93]. Patterns of high intercorrelations will be used to create a concise pupillometric battery of LC-NE functioning (~15 min). BPS and SEPR are reliably assessed in few oddball trials (k=20).

### Expected Outcome and Study Hypotheses

A successful task manipulation will be indicated by a higher SEPR in oddball versus standard trials (ie, sensory selectivity) and declining BPS across standard trials (ie, habituation). We expect convergent validity with moderate-to-high correlations between the auditory oddball SEPR and the pupillometric measures in the validation tasks (including SEPR to a change from regularity to irregularity, SEPR to visual oddball stimuli, and SEPR before target detection). We expect convergent validity in the hetero-method analyses, whereas we expect divergent validity with cognitive and language development.

### Risk Assessment

We only include paradigms that are feasible in young children. This shall ensure a sufficient completion rate in preschoolers (>80%). The concurrent presentation of visual stimuli to maintain attention during the auditory oddball task may impede the SEPR. However, we recently showed in independent data that visual presentation during an auditory oddball task still induces distinct task-evoked pupillary responses.

### Work Package 2 (Objective 2): Association of LC-NE Functioning With Correlates of Psychopathology

#### Task 2.1: LC-NE Functioning Corresponds to Early Dispositional Risks for Psychopathology

##### Participants

A sample of 140 preschool-aged children will be recruited in accordance with the general recruitment strategy of the study.

##### Sample Size and Power

A power analysis based on an LMM indicates that a sample of 140 participants provides power of *β*=.84 to detect a moderate correlation (b=0.3) between LC-NE functioning and dispositional risks.

##### Experimental Design

Cross-sectional analysis of combined pupillometry, EEG, and behavioral and cognitive assessments.

##### Neurophysiological Measures

The validated pupillometric battery, as described in Objective 1, will be used to assess BPS and SEPR across multiple tasks. In a concurrent EEG, the ERP of ERN will be assessed using an age-appropriate Go/No-Go task [50]. Neurocognitive paradigms of irritability will be examined using a dot-probe task [95] and a frustrative reward learning task [96].

##### Cognitive Measure

To assess the verbal and cognitive abilities, we will conduct 2 subtests from the WPPSI [93].

##### Behavioral Measures

Temperament traits will be measured via caregiver reports with the Behavioral Inhibition Questionnaire (BIQ) [83] and Affective Reactivity Index (ARI) [84]. Negative emotionality will be assessed via Child Behavior Questionnaire-Very Short Form (CBQ-VSF) [85]. The 3 transdiagnostic factors of psychopathology will be assessed with the Child Behavior Checklist (CBCL 4‐18) [82].

##### Statistical Analysis Plan

We will apply LMM analysis with participants as random intercepts. These will allow us to explore associations between variables on a per-task level and control for potential covariates (age, gender, and IQ).

##### Expected Outcome and Study Hypotheses

Behavioral inhibition (BIQ score) is expected to correlate positively with both BPS and SEPR in the pupillometric battery of LC-NE functioning. In contrast, irritability (assessed via ARI score) is expected to show differential profiles of LC-NE functioning. We expect high irritability scores and high SEPR to be associated with higher internalizing symptoms, while high irritability scores and low SEPR will be associated with higher externalizing symptoms—these expected outcomes are supposed to match ERN profiles [101]. Furthermore, in the neurocognitive paradigms, participants with high-irritability-low-SEPR profiles are further expected to show increased attentional bias toward threat cues (threat perception) and decreased attentional shifts during frustration (frustrative reward learning). These patterns would suggest a subgroup with elevated risk for conduct disorders. We will further explore LC-NE functioning in children with both high irritability and high behavioral inhibition, who are expected to exhibit higher negative emotionality and an increased BPS as an index of an elevated LC-NE tonic activity.

##### Risk Assessment, Participant Burden, and Ethical Considerations

This task is associated with the highest participant burden due to its combined EEG assessments and neurocognitive task demands. To mitigate this, we will provide additional breaks for the participants and offer food and drinks between assessments. We assume a higher attrition rate for this task compared to other tasks, estimated at approximately 30%. Thus, this task is designed as more optional, ensuring that potential dropouts will not compromise subsequent study objectives.

### Task 2.2: LC-NE Function Mediates the Association Between Childhood Adversity and Transdiagnostic Psychopathology

#### Participants

A sample of 300 preschool-aged children (aged 4‐6 years) will be recruited in accordance with the general recruitment strategy of the study.

#### Sample Size and Power

A power analysis based on a structural equation model indicates that a sample of 300 children provides a power of *β*=.80 to detect a moderate path coefficient (a=0.2) between latent variables of LC-NE functioning and psychopathology.

#### Experimental Design

Cross-sectional analysis of combined pupillometry, behavioral, and cognitive assessments will be used.

#### Neurophysiological Measures

The validated pupillometric battery, as described in Objective 1, will be used to assess BPS and SEPR across multiple tasks.

#### Cognitive Measure

To assess the verbal and cognitive abilities, we will conduct 2 subtests from the WPPSI [93].

#### Behavioral Measures

Transdiagnostic psychopathology factors (externalizing, internalizing, and p-factor) will be assessed with CBCL 4‐18. In addition, we will assess childhood adversity based on multiple sources. Specifically, a caregiver interview that captures adverse childhood experiences and other psychosocial domains (ACE+) [40], a respective child interview [86], validated child-report measures of adversity [88,89], and a report by the respective clinician from the outpatient clinic for those children recruited via the outpatient recruitment leg. This assessment approach will provide an index of cumulative adversity or will be differentiated into specific adversity domains.

#### Statistical Analysis Plan

Structural equation models will be applied to estimate the associations between the latent constructs (LC-NE functioning, transdiagnostic psychopathology, and childhood adversity). A best-fit path model will be used to reduce the complexity of the structural equation in the longitudinal study of Objective 3. A latent mediation model, in which LC-NE functioning is expected to mediate an association between previous childhood adversity and transdiagnostic psychopathology, will be applied. Independent models for BPS, SEPR, and each transdiagnostic factor will be estimated. Classification approaches will be applied in exploratory analyses to identify LC-NE function profiles in subgroups of psychopathology.

#### Expected Outcome and Study Hypotheses

We hypothesize associations between retrospective childhood adversity, LC-NE function, and transdiagnostic psychopathology. For example, lower LC-NE tonic activity is expected to represent a resilience to the negative impact of childhood adversity on psychopathology. In addition, we expect profiles of LC-NE functioning that are associated with patterns of transdiagnostic psychopathology; BPS is expected to positively correlate with the p-factor, whereas increased and decreased SEPR are expected to positively correlate with internalizing and externalizing symptoms, respectively.

#### Risk Assessment, Participant Burden, and Ethical Considerations

We anticipate that some children will require immediate support, and we will use our clinical infrastructure to provide further case management. In addition, reports of adversity will be evaluated according to our in-house reporting guidelines, which align with the German child protection laws. This includes evaluation in a joint discussion and may result in reporting to child protection authorities when imminent danger is identified.

### Work Package 3 (Objective 3): Prospective Effect of LC-NE Functioning in a Developmental Model of Vulnerability and Stress

#### Task 3.1: LC-NE Functioning Predicts Later Transdiagnostic Psychopathology.

##### Participants

The study will follow the sample of 300 preschool-aged children, initially assessed in Task 2.2 at 4‐6 years of age across 3 time points.

##### Sample Size and Power

**Figure 6.**
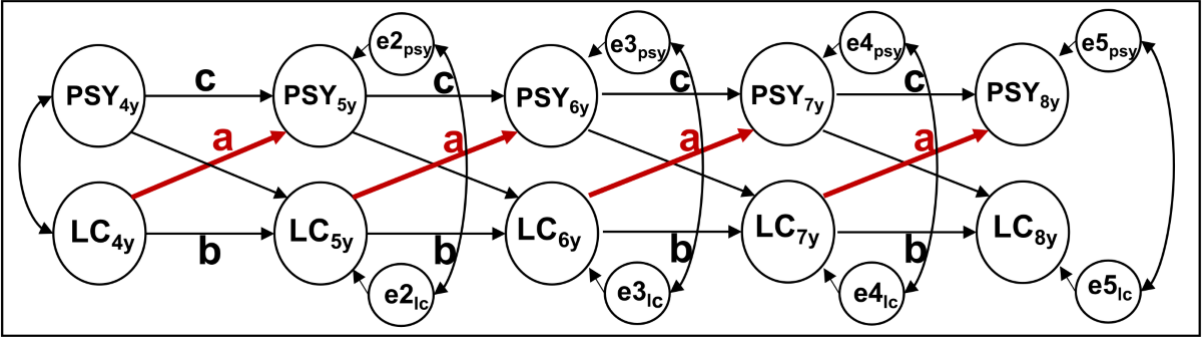
Autoregressive cross-lagged panel model to investigate prospective effects (**A**) of LC-NE functioning on psychopathology (PSY). b and c: autoregressive effects; e2i, e3i, e4i, and e5i: time-specific measurement errors; LC: locus coeruleus; PSY: psychopathology.

For the core longitudinal study with a cross-lagged panel model, power is *β*=.82 for 300 participants to detect a moderate cross-lagged effect of LC-NE functioning on psychopathology (a=0.2; see Figure 6).

##### Experimental Design

The study uses an accelerated longitudinal design with 3 assessment waves and time points: the baseline or initial assessment as part of Task 2.2 (T1; aged 4‐6 years), a 1-year follow-up (T2; aged 5‐7 years), and a 2-year follow-up (T3; aged 6‐8 years). At each time point, we assess neurophysiological (pupillometry), behavioral, and cognitive measures.

##### Neurophysiological Measures

At each time point, the validated pupillometric battery, as described in Objective 1, will be used to assess BPS and SEPR across multiple tasks.

##### Cognitive Measure

To assess the verbal and cognitive abilities, we will conduct 2 subtests from the WPPSI [93].

##### Behavioral Measures

At each timepoint assessment of the behavioral measures, as described in Task 2.2, will include caregiver questionnaires (CBCL 4‐18) and the multiple-source assessment of childhood adversity (see Table 1).

##### Statistical Analysis Plan

The accelerated longitudinal design allows estimation of an autoregressive cross-lagged panel model that will quantify the effect of previous LC-NE functioning (BPS and SEPR) on later transdiagnostic psychopathology factors (CBCL) across the age of 4-8 years (see Figure 6). We will control for potential cohort effects (initial age: 4 vs 5 vs 6 years) by including cohort as a random intercept. Each model fit will be evaluated by the predefined indices of CFI, TLI, RMSEA, and SRMR.

##### Expected Outcome and Study Hypotheses

This core of LOCUS-MENTAL will be informed by the outcome of task 2.2. We expect (see Figure 1) that higher BPS as an index of LC-NE tonic activity predicts a higher p-factor. Increased and decreased SEPR as an index of LC-NE phasic activity is expected to predict higher internalizing and externalizing symptoms, respectively. This would predict future psychopathology based on pupillometric markers. The stability of the investigated constructs is controlled for by autoregressive effects. The structural equation models will be explored for the covariate effects of childhood adversity, previous psychopathology, and received clinical support (eg, in the context of the outpatient clinic for early detection).

##### Risk Assessment

A major risk is a low retention rate. A meta-analysis identified a mean retention rate of 73.5% and identified strategies to increase it [102]. This includes barrier reduction by including remote data collection (eg, offer data collection via phone and online), tracing strategies by locator documents with contact information, and community-building by sending participants thank-you, birthday, and holiday cards. We will implement these strategies in LOCUS-MENTAL to maximize our retention rate while maintaining ethical standards.

### Task 3.2: LC-NE Functioning Mediates the Effect of Stress Responses on the Development of Transdiagnostic Psychopathology

#### Participants

Participants in this study will comprise a subsample of 5‐6-year-olds (n=160) at one randomly selected timepoint (T2 or T3) from the large longitudinal cohort (Task 3.1).

#### Sample Size and Power

A power analysis using a bivariate latent growth curve model indicates a power of *β*=.82 for 160 participants to detect a moderate path coefficient (b=0.2) of cortisol reactivity on changes in psychopathology.

#### Experimental Design

This task uses a cross-sectional assessment of endocrine stress responses within the longitudinal study.

#### Measures

Endocrine stress responses provide a more proximate measure of stress compared to childhood adversity. We will assess stress responses through hair cortisol levels [103] and salivary cortisol measurements following a developmentally appropriate social stress test [94]. Hair cortisol levels will quantify the retrospective stress levels, while salivary cortisol will quantify the acute stress response.

For hair cortisol, we will collect 3 centimeters in length of scalp-nearest hair; this will contain cortisol information over the preceding 3 months. Salivary cortisol will be collected via fiber chewing before and after a social stress test (after 30-40 minutes). In the social stress test, the children are instructed to match stickers to animals to win a prize. Time pressure will be applied by a commenting experimenter and a toy traffic light that turns red and emits a buzzer sound [94,104]. Contractors will process salivary (Daacro) and hair (Dresden Lab service) samples.

#### Statistical Analysis Plan

The cortisol measures will be applied in a mediation model to examine LC-NE functioning in the association of stress response and psychopathology. We will further quantify the effect of LC-NE functioning in the association of childhood adversity and stress response.

#### Expected Outcome

We expect a positive association between cortisol measures and psychopathology. Based on our model (Figure 1), we assume BPS and SEPR to mediate this direct effect. Higher BPS and SEPR are expected to explain an increased effect of hair cortisol levels and salivary cortisol response on the internalizing symptoms and the p-factor. Lower BPS is expected to be a protective factor on the negative impact of stress on psychopathology, which could represent a mechanism of resilience. Based on our preliminary work (see above), we expect an attenuated downregulation of salivary cortisol in response to the social stress test (ie, inefficient acute stress regulation) to be associated with higher internalizing symptoms and a higher p-factor. We expect this effect to be mediated by BPS as an index of LC-NE tonic activity.

#### Risk Assessment

Childhood adversity might not be disclosed by caregivers if they are also the perpetrators, while child reports on adversity might be unreliable. To mitigate the underreporting biases, we will implement the multiple-sources approach to adversity. In addition, any reports on adversity will be evaluated according to our in-house reporting guidelines and may lead to further actions.

### Work Package 4 (Objective 4): Development of an Objective Tool of Risk Prediction in the Preschool Age Based on the Pupillometric Measures of LC-NE Functioning

#### Participants

The full sample was recruited during work packages 2 and 3.

#### Sample Size

The full sample consists of the 300 participants assessed in task 2.2.

#### Experimental Design

The findings of Objectives 2 and 3 will be used to enrich a probabilistic model in the full sample (n=300) that assigns individual risk based on LC-NE functioning in the preschool age. This will be evaluated as a tool of quantitative risk prediction for future psychopathology. We will operationalize risk probability within a normative model of transdiagnostic psychopathology [105]. A reference cohort will be defined from a subsample of participants that did not develop clinically relevant transdiagnostic psychopathology. This cohort will be applied to estimate a normative development model (ie, output) as a function of LC-NE tonic and phasic activity profiles. It defines a range of LC-NE functioning variation that is associated with a low risk. The model can then be applied to withheld data, that is, individuals who did develop transdiagnostic psychopathology. This allows one to quantify a numeric deviation of LC-NE functioning from normative ranges. The numeric deviation is then applied as a predictor of psychopathology in linear models that represent an individual risk prediction. The performance of individual risk prediction based on LC-NE functioning will be compared to the predictive validity of available covariates, confirmed by v-fold validation, and implemented in the outpatient clinic for external validation in independent data.

#### Expected Outcome

We expect LC-NE functioning to provide added predictive validity, with an increase in explained variance compared to previous psychopathology, childhood adversity, and cognitive ability. We assume a moderate accuracy of the normative model and substantial associations of normative range deviations with transdiagnostic psychopathology. We further expect that the spatial direction of the range deviation within the normative model differentiates the factors of transdiagnostic psychopathology. The simplicity of the model emphasizes its feasibility for use in clinical settings. The final task of LOCUS-MENTAL will provide a tool for quantitative risk prediction in at-risk populations of preschoolers.

#### Risk Assessment

We acknowledge that LC-NE functioning is not the only determinant in the complex development of psychopathology, but given this, the time is ripe to test a promising neurophysiological mechanism as an objective predictor that improves the assessment of clinical risk over the current practice of subjective evaluation.

### Ethical Considerations

The study was approved by the Ethical Committee (reference number: 2024‐2160) and the Data Protection Officer of the Medical Faculty of Goethe University Frankfurt. To date, no adverse events have been reported by any comparable study, and thus we expect no risks nor harm to our participants. The study is planned and will be conducted and analyzed according to Good Scientific Practice (GSP) guidelines, the Declaration of Helsinki, and the European Data Protection Directive (DSGVO). Study participation is on a voluntary basis, and participants can withdraw from study participation at any time without any adverse consequences concerning their medical or psychiatric treatment in our clinic. Participants will also receive monetary compensation for their time. All relevant information regarding data protection and the voluntary basis of participation will be communicated to participants and their legal caregivers verbally, as well as in the form of written informed consent forms. We also obtain verbal assent from the preschool participants. The child’s right to withdraw at any time will be prioritized over parental consent. During assessments, the child will be closely monitored by the experimenter that is present at all times, and signs of acute distress (such as crying, physical withdrawal, or verbal expression of a desire to stop) will lead to termination of the assessment. In cases of suspected or disclosed adversity, the research team will inform the principal investigator immediately (licensed psychotherapist for children), who offers a voluntary counseling appointment to discuss potential adversity with the family and the child. In line with German child protection laws and in accordance with our clinical guidelines, the quantity and quality of suspected adversity might result in further support by our department or even a report to authorities (eg, imminent danger of ongoing adversity). We acknowledge that the development of a risk assessment tool could potentially lead to stigmatization and misuse. With our data management plan outlined below, we made sure that individual risk prediction cannot be associated with personally identifiable information. We further refrain from any nonscientific use of the prognostic risk marker. The rights to the information of prognostic risk remain with the participants in accordance with DSGVO. We are confident that a prognostic risk marker also provides the opportunity to shift from a health care system that focuses on intervention after manifestation of psychopathology to a focus on targeted prevention. Children at an elevated risk for mental health problems as identified by LOCUS-MENTAL could receive further case management at our outpatient clinic, where we decide on useful next steps on a case-by-case basis with evidence-based treatments. For example, caregivers of children at risk could be offered the opportunity to receive parent trainings that are established in the treatment of mental health problems in children. This may aid in the early prevention of the initial manifestation of psychopathology. Our newly established outpatient clinic for early detection could be the ideal environment to use risk assessment information for positive clinical outcomes.

LOCUS-MENTAL applies a noninvasive neuroimaging methodology that allows indexing neuronal activity by video-based pupillometry. Previous work showed feasibility and established this methodology in clinical samples of children. In addition, we apply further noninvasive assessments with electroencephalography. These measures introduce a burden for the participants and can induce a state of irritability but do no harm. This also applies to the application of an age-appropriate social stress test. The test induces a temporary impression that the child’s performance was insufficient in a task, which has been associated with a cortisol release response that needs to be quantified to achieve the objective of task 3.2. The social stress test thus includes a comprehensive disclosure strategy after the test to alleviate any negative effects. In task 3.2, we further collect tissue as hair samples (cutting off hair strands) and saliva samples (chewing an artificial fiber). These methods of tissue collection are also entirely non-invasive. The tissue samples will be processed by experienced contractors and subsequently destroyed. In the project, we further assess personally identifiable information that is handled according to the descriptions in section 2.4. We expect that this study does not involve an immediate risk of yielding knowledge, products, or technology that could intentionally be misused to cause substantial harm.

Questionnaire and interview data will be collected via our on-premises online tool according to the European General Data Protection Regulation (GDPR). We further do video-based eye tracking, apply EEG, and collect biological samples. Hair samples will be analyzed by Dresden Labservice with online solid-phase extraction in liquid chromatography-tandem mass spectrometry. Saliva samples will be analyzed by Daacro Contract Research with the ELISA Salimetrics Assay in a double estimation. Metadata will be generated by a printed case report form. Data are transferred to electronic databases that allow for automated quality controls and sanity checks. Eye tracking and EEG data will also receive quality control by peer-reviewed preprocessing pipelines. We established standard operating procedures to document research data, personnel will be trained in GSP, and all data handling will be in accordance with the GDPR. We differentiate between person-identifying information and research data that are separated in different networks, both behind the clinic’s firewall. The data can only be combined by a person identification code that is restricted in access to authorized personnel and stored on the clinical network. This is separated from a pseudonymized research network. All databases run on a Microsoft Access front- and back-end with an Active Directory system. Data are stored on on-premises servers with backups in a different building. After the project’s end, anonymized research data will be made available according to Open Science standards and archived for 10 years. The author (IT) will administer the project-specific databases. PhD students will have read and write rights with log protocols. A dedicated database manager with a permanent position is the administrator of all databases.

The LOCUS-MENTAL study uses targeted strategies across its work packages to manage missing data and ensure robust longitudinal findings. Work package 1 focuses on feasibility measures by using passive and child-friendly paradigms to achieve a projected completion rate of over 80%. Statistical analysis will exclude children that have per-task data validity rates of less than 50% or more than one missing task. The auditory oddball task is a marker task that is required to be included in the statistical analysis. We established standardized procedures of pupillometry data preprocessing to address within-trial missing data due to inattention. Work package 2 addresses high-burden EEG assessments by designating them as “more optional” in the context of the overall project, which allows for an expected 30% attrition rate without hindering subsequent study objectives. For the longitudinal assessments in work package 3, the study implements retention strategies like online data collection and locator documents. In addition, the statistical framework of work package 3 includes LMM and structural equation modeling to handle intermittent missingness across time points. Questionnaire data on an item level with per-measure missing rates up to 20% will be addressed by Multiple Imputation by Chained Equations (MICE) that preserves statistical power under the missing at random assumptions. However, children with increased irritability or increased burden may be more likely to drop out (ie, missing not at random or MNAR). Thus, pattern mixture models will be applied as a sensitivity analysis to determine if the developmental trajectories of LC-NE functioning are biased by specific attrition patterns. In addition, Inverse Probability Weighting (IPW) in the longitudinal data analysis can be used to adjust for non-random attrition by weighting the remaining participants based on baseline characteristics (eg, socioeconomic status or initial psychopathology scores) that predict dropout. Missing bio samples will be addressed by case-wise exclusion in the specific subanalysis. Finally, work package 4 uses v-fold validation to evaluate the predictive stability of the risk prediction tool.

## Results

The project was launched upon funding in November 2024. The validation phase (work package 1) began participant enrollment in August 2025. As of February 2026, 82 participants have been assessed. Recruitment is ongoing, with a target of 90 participants by the end of February 2026. Data collection for the validation phase will be followed by comprehensive analysis of the psychophysiological and behavioral data. Results from this phase are intended for publication in 2026. Based on the validated paradigms, the core accelerated longitudinal study (work package 2) is scheduled to commence in April 2026. This longitudinal component will follow a cohort of 300 preschool-aged children across 3 annual assessment waves till March 2029. The entire LOCUS-MENTAL project is designed as a 6-year study. Final data analysis, synthesis of findings, and dissemination for the full longitudinal cohort are planned to conclude by the end of 2030.

## Discussion

### Principal Findings

The LOCUS-MENTAL protocol outlines a longitudinal study design with the primary aim to establish LC-NE functioning as a neurobiological predictor of later psychopathology in childhood. We anticipate that the validation phase (Objective 1) will demonstrate convergent and discriminant validity of the pupillometric markers BPS and SEPR with sufficient reliability. By developing and validating a child-friendly, pupillometry-based tool, this aims to bridge a critical gap between neurobiological mechanism and clinical risk prediction, which is based solely on behavioral observation. Accordingly, this is a prerequisite to ensure the method sensitivity and specificity. This would provide a concise test battery that assesses a neurobiological mechanism that could be implemented in future clinical studies capturing a potential biomarker of psychopathology in early childhood. Furthermore, we hypothesize that BPS and SEPR will cross-sectionally differentiate between transdiagnostic risk profiles, such as irritability and behavioral inhibition, and serve as a mediator between childhood adversity and psychopathology (Objective 2). This would provide an underlying neurobiological basis for temperament dimensions that are also based on caregiver reports. In the longitudinal data, as a core outcome, we expect to reveal baseline LC-NE functioning at age 4‐6 years to predict the development of psychopathology at school age (Objective 3). This central component of the LOCUS-MENTAL project would provide causative evidence for an impact of LC-NE functioning on transdiagnostic mental health conditions. These findings will be used in forming a normative model as a pupillometry-based risk prediction tool, establishing a key neurobiological susceptibility mechanism that mediates the impact of early adversity on psychopathology (Objective 4). This will allow a translation of the experimental findings to clinical practice. For example, children associated with an elevated risk for psychopathology based on LC-NE functioning could be supported by evidence-based treatments that are effective before the onset of a mental health condition. This would emphasize a targeted prevention approach. We conclude that the identification of a neurobiologically grounded risk marker could transform our mental health infrastructure from interventions that are applied years after the onset of mental health problems to early and targeted support. Ultimately, this aims to reduce the lifetime burden of mental health problems.

### Strengths

LOCUS-MENTAL focuses on a critical developmental window, the preschool period (aged 4‐6 years), a key time for the emergence of transdiagnostic risk factors, offering a unique opportunity for targeted prevention. This developmental period has been largely neglected in experimental research on lifetime mental health, as preschoolers have limited capabilities to follow task instructions. In this project, this is addressed by the application of feasible pupillometry as a primary method that is feasible in young and burdened children. The study provides a robust design, combining an initial multitrait-multimethod validation phase with a subsequent large-scale accelerated longitudinal study. This design strengthens internal validity through paradigm optimization and allows for examination of causal pathways and developmental trajectories over time.

### Limitations

The novelty of the paradigms for preschoolers is a limitation since established pupillometric paradigms for assessing specifically LC-NE functions have not been established in young children. The dedicated validation phase (working package 1) is specifically designed to address this issue. Another limiting factor is the longitudinal study design. This design comes with the risk of high participants’ attrition, which may affect the final statistical power. To address this, the study will use retention strategies (eg, regular contact, family-friendly appointment scheduling, and compensation) and will use appropriate statistical methods to handle missing data.

### Future Directions

If the pupillometric markers are validated as successful predictors, future research could focus on clinical implementation. The natural next step would be the development of a pupillometry tool that can be applied outside the lab, for which head-mounted devices with built-in eye trackers are a promising avenue. Ultimately, this would deliver a child-friendly and low-cost automated screening tool for pediatricians or for educational settings.

### Dissemination Plan

The results of the study will be disseminated through peer-reviewed publications in high-impact journals. Findings will be presented at national and international conferences. Following Open Science principles, data analysis will be made available in online repositories, while deidentified data can be shared upon reasonable request in line with data privacy considerations. In addition, we engage in active patient and public involvement by media outreach and promoting our research on social media and our Mind-Child lab web page.

## Supplementary material

10.2196/89237 Checklist 1 Completed STROBE (Strengthening the Reporting of Observational Studies in Epidemiology) checklist for the LOCUS-MENTAL longitudinal study protocol.

10.2196/89237 Peer Review Report 1 Peer-review report by Emmy-Noether Program Review Panel, Excellence Research Funds of the German Research Foundation (DFG; Germany).

## Abbreviations

ACC: anterior cingulate cortex
AGT: Adaptive Gain Theory
ARI: Affective Reactivity Index
BIQ: Behavioral Inhibition Questionnaire
BPS: baseline pupil size
CBCL: Child Behavior Checklist
CBQ-VSF: Child Behavior Questionnaire-Very Short Form
DSGVO: European Data Protection Directive
EEG: electroencephalography
ERN: error-related negativity
ERP: event-related potential
GDPR: General Data Protection Regulation
GSP: Good Scientific Practice
HPA: hypothalamic-pituitary-adrenal
IPW: Inverse Probability Weighting
LC: locus coeruleus
LC-NE: locus coeruleus-norepinephrine
LMM: linear mixed models
MHD: mental health disorder
MICE: Multiple Imputation by Chained Equation
RAND20: random 20
REG 10: regular 10
SEPR: stimulus-evoked pupillary response
STROBE: Strengthening the Reporting of Observational Studies in Epidemiology
WPPSI: Wechsler Preschool and Primary Scale of Intelligence
