# Correction: Locus-Coeruleus Norepinephrine Functioning as a Predictor of Childhood Mental Health (LOCUS-MENTAL): Protocol for a Longitudinal Study

**DOI:** 10.2196/100635

**Published:** 2026-07-09

**Authors:** Iskra Todorova, Paula R Neubauer, Nico Bast

**Affiliations:** 1 Department of Child and Adolescent Psychiatry, Psychosomatics and Psychotherapy, Goethe-University, University Hospital Frankfurt, Deutschordenstraße 50, Frankfurt am Main, Hessen, 60528, Germany, 49 696301 ext 5279

In “Locus-Coeruleus Norepinephrine Functioning as a Predictor of Childhood Mental Health (LOCUS-MENTAL): Protocol for a Longitudinal Study” [1], the authors noted one error.

In the Introduction subsection Pupillometry Reliably Measures LC-NE Functioning in Clinical Settings, the sixth sentence has been amended.

The phrase previously appeared as:

*In a rodent model, an LC-NE stimulation predicted pupil dilation after 200‐300 milliseconds [34].*

The phrase has been revised to:

*In a nonhuman primate model, an LC-NE stimulation predicted pupil dilation after 200‐300 milliseconds [34].*

The correction will appear in the online version of the paper on the JMIR Publications website, together with the publication of this correction notice. Because this was made after submission to PubMed, PubMed Central, and other full-text repositories, the corrected article has also been resubmitted to those repositories.
