# Supplementary material for: Locus-Coeruleus Norepinephrine Functioning as a Predictor of Childhood Mental Health (LOCUS-MENTAL): Protocol for a Longitudinal Study
Source: JMIR Res Protoc. 2026 Apr 20;15:e89237. doi: 10.2196/89237 (PMC13095051; doi:10.2196/89237)
Supplement: Peer Review Report 1 [file resprot-v15-e89237-s002.pdf]

Stellungnahmen zum Antrag

Projekt: „LOCUS-MENTAL - Das Locus-Coeruleus-Norepinephrine-System als Prädiktor mentaler Gesundheit im Kindesalter“ (BA 7327/6-1)

Antragstellende Person: Herr Dr. Nico Bast, Frankfurt

Gutachten 1:

“

1. How would you evaluate the soundness of the preliminary work, the quality of publications (please refer to the Guidelines for Publication Lists) and the **qualifications of the applicant** – both in general and in terms of the proposed project?

The preliminary work is extremely useful in highlighting the feasibility of the research (including recruitment of participants and collection of all measures) and the validity of the pupillometry measures. The applicant clearly has the appropriate qualifications and experience to successfully conduct this project. The applicant has an excellent publication track-record.

Does the applicant have the ability to independently lead a junior research group? How would you assess the applicant's chances of being appointed to a professorship after the independent junior research group ends?

The applicant already leads a small research team, and as such they have already proven that they can independently lead a junior research group. The high quality of the proposed work provides some confidence in the applicant's chances of being appointed to a professorship.

2. How would you assess the **quality of the project**, especially with regard to originality and the anticipated contribution to knowledge?

The applicant proposes a series of studies that investigate pupillometry measures (as indices of LC-NE functioning) as early indicators of risk for mental health problems in children. I found the proposed research to be of excellent quality. The series of proposed studies build on each other, and will result in a comprehensive assessment of the utility of LC-NE functioning measures to predict risk for mental health problems in children. The proposed work is original and will result in advanced knowledge of markers of LC-NE functioning (pupillometry measures) and their relation to mental health risk.

I have one issue with how the proposed work is pitched. The applicant proposes that LC-NE functioning may explain a differential susceptibility to psychosocial adversity that underlies dispositional risk for mental health problems. However, they do not propose to test differential susceptibility, which would require the testing of interactions between LC-NE functioning and adversity in the prediction of mental health symptoms (i.e., moderation analysis).

I suspect that the applicant did not mean that they want to test differential susceptibility; rather, I suspect they want to test LC-NE functioning as a mechanism linking adversity and mental health problems, which is perfectly appropriate. However, they should be careful and precise with their framing of their project.

3. To what extent do the **objectives and work programme** convincingly reflect clear working hypotheses and an appropriately distinct topic? Please comment on the strengths and weaknesses of the planned investigations, the suitability of the methods and the appropriateness of the schedule. How do you rate the explanations on the handling of research data?

The objectives and work programme do reflect clear working hypotheses. General strengths include the ability to recruit from a clinic that the applicant works at, the availability of a testing lab that the applicant has set up, and the accelerated longitudinal design employed for some research questions. The methods are all generally suitable and the schedule seems feasible.

Regarding 3.1, it was unclear to me why this question is to be addressed with cross-sectional data. Longitudinal data for mediation analyses are clearly superior. For 3.2, cortisol measures will be assessed at t2 or t3, even though they will be used as the predictor. This seems suboptimal; it is unclear why cortisol is not measured at t1 such that the IV, mediator and DV are spaced in time.

I would have liked to see more details of the approach for oversampling children with internalizing problems. Also, it is unclear if children with low levels of symptoms can be recruited from the clinic given they are presumably admitted due to existing issues.

A further general comment: what is the rationale for age 4-8? Many mental health problems emerge during adolescence so there is a question of whether there will be enough variance in mental health problems at age 8.

4. How would you assess the **work and research environment**? Is the host institution suitable?

Excellent.

5. Applicants in the Emmy Noether Programme have the option of naming more than one potential host institution in the proposal. A final decision does not have to be made until after the proposal is approved. Therefore, please comment on all institutions listed.

N/A

6. Please provide a **clear recommendation** as to whether the proposal should be approved. If so, comment on whether the requested funds are justified and reasonable in relation to the proposed project, making recommendations for adjustments to the budget as necessary.

I highly recommend approval of the proposal. Despite my concerns about the theoretical model, and of appropriate design for mediation analyses, I have confidence that the applicant will deliver significant research outcomes that have the potential for clinical utility."

<u>Gutachten 2:</u>

1. Ist die Fähigkeit zur selbständigen Leitung einer wissenschaftlichen Nachwuchsgruppe gegeben? Wie beurteilen Sie die Berufungschancen nach Abschluss der Nachwuchsgruppe?

Dr. Nico Bast is highly qualified for conducting the proposed research. As I stated in my previous evaluation, he has developed an innovative method of using pupillometry in child samples and he published important results on the use of the method. Preliminary data are presented that support the hypothesis and that demonstrate the feasibility of the proposed studies. Taken together, the application convincingly demonstrates the high expertise of the applicant in leading and conducting the proposed research. I had previously commented that the expertise of Dr. Bast relates to autism in children and that he needs to demonstrate expertise to conduct research on adversity in preschool-aged children. In the revised application he added safety measures and implemented an inhouse reporting guideline based on the child protection law (§4 KKG).

2. Wie beurteilen Sie die Qualität des Vorhabens, vor allem hinsichtlich Originalität und erwartetem Erkenntnisgewinn?

As stated in my previous evaluation, I think that the proposed research, with no doubt, demonstrates exceptionally high levels of both quality and innovation. I also stated previously that the proposed research has the potential to provide an objective neural risk marker tool that can be broadly applied in child populations, which is highly promising. The relevance and potential to enhance knowledge and improve diagnostic practice and prospective prediction of risk trajectories in child psychiatry is high.

3. Inwiefern überzeugen Ziele und Arbeitsprogramm hinsichtlich der Klarheit der Arbeitshypothesen und einer sinnvollen Eingrenzung der Thematik? Benennen Sie bitte Stärken und Schwächen der geplanten Untersuchungen, die Angemessenheit der Methoden und des Zeitplans. Wie bewerten Sie die Ausführungen zum Umgang mit Forschungsdaten?

In my previous evaluation, I stated that the aims and methods demonstrate a high level of innovation and scrutiny. I had comments and questions related to the design and methods.

1) The applicant clarified the definition of ‘children of concern’. I am uncertain whether my questions regarding the inclusion of control children as well as different groups sizes across work packages were addressed.

2) My core question concerned whether LC-NE activity is considered a trait-like disposition that predicts emergence of symptoms across diagnostic categories and that moderates susceptibility

to stress, i.e. emergence of symptoms after adversity. Based on available literature I rather considered the LC-Ne system as modifiable by early stress and, hence, rather as a mediator of risk. The applicant now reframed the model and conceptualizes the LC-NE system (and changes by stress) as a mediator.

3) I asked for associating LC-NE function with personality traits and temperament. This has now been added. This was in my reasoning relevant to strengthen the idea that LC-NE function is a moderator/disposition, see above discussion, but adding these measures and analyses might still be informative.

4) The relevance of the LC-NE system across diagnostic categories is now sufficiently discussed.

5) My concerns regarding the suitability of the stress task and habituation effects have been addressed in a very elaborated fashion and are now sufficiently addressed. The response includes a changed design and methodology, by a) limiting the application of the social stress task to preschool-aged children, by b) applying the “social stress test” only one, and not repeatedly within participants, randomized at t2 or t3, and by c) adapting the statistical modelling.

6) I previously had a question regarding the validity of parent reports and disclosure biases. This concern was sufficiently addressed. The applicant now uses multiple informants and self reports.

4. Wie schätzen Sie Arbeitsmöglichkeiten und das wissenschaftliche Umfeld ein? Ist die gastgebende Einrichtung geeignet?

The work environment and host institution are ideal and I am convinced that the institution will fully support the Emmy Noether group. The Department of Child and Adolescent Psychiatry at Goethe Universität Frankfurt is an ideal site to conduct the research. It provides access to large numbers of children of concern and the necessary clinical embedding of such as study. The applicant has demonstrated convincingly that he can productively work in this environment and with his colleagues.

5. Antragstellende im Emmy Noether-Programm haben die Möglichkeit, in ihrem Antrag mehrere aufnehmende Institutionen anzugeben. Eine endgültige Entscheidung muss erst nach der Bewilligung getroffen werden. Bitte nehmen Sie daher zu allen angegebenen Institutionen Stellung.

Not applicable.

6. Bitte formulieren Sie ein eindeutiges Votum für oder gegen eine Förderung. Bitte machen Sie bei einem Votum für eine Förderung auch einen konkret ausdifferenzierten Mittelvorschlag. Berücksichtigen Sie dabei gegebenenfalls, ob die beantragten Mittel angemessen sind.

I recommend the Emmy Noether Programme application for funding due to its high innovation, scientific rigor, and high potential for direct translation into practice. The budget seems adequate."

Stellungnahme des Fachkollegiums:

"Zu diesem überarbeiteten Emmy Noether-Antrag liegen zwei Gutachten vor, die den Antrag eindeutig zur Förderung empfehlen. Das erste Gutachten ist von einer Person geschrieben, die den vorherigen Antrag nicht begutachtet hatte. Dieses Gutachten lobt die Vorarbeiten als extrem nützlich und den Antragsteller als hervorragend qualifiziert, um das Projekt und eine Nachwuchsforschungsgruppe zu leiten. Ein Kritikpunkt nennt das Gutachten in Bezug auf das theoretische Modell: Es sei nicht klar, ob das LC-NE-System eine Disposition darstelle oder einen Mediator des Zusammenhangs zwischen Childhood Adversity und Psychopathologie. Das Gutachten nimmt zwar an, dass es sich um einen entsprechenden Zusammenhang handelt, wünscht sich aber eine klarere Formulierung. Der Kritikpunkt war auch bereits in der ersten Begutachtungsrunde aufgekommen und wurde im Response-Letter des Antragstellers auch klar adressiert. Hier stellt der Antragsteller dar, und dies ist auch dem Antrag zu entnehmen, dass das LC-NE-System einen Mediator darstellt. Dies wird auch im zweiten Gutachten so festgestellt, das von der Person stammt, die den Kritikpunkt in der ersten Begutachtung vorgebracht hatte und dies nun als zufriedenstellend klargestellt ansieht. Aus Sicht des Fachkollegiums ist daher dieser Punkt nicht kritisch. Einen weiteren Kritikpunkt von Gutachten 1 an der nicht durchgehend durchgeführten Cortisol-Messung zu T1 kann das Fachkollegium ebenfalls nicht vollständig teilen - diese Planung geht auf die Kritik aus der vorherigen Begutachtung zurück, bei der mögliche Habituationsprozesse diskutiert wurden. Es ist dem Antragsteller hervorragend gelungen, das Design so anzupassen, dass trotzdem längsschnittliche Analysen durchgeführt werden können. Auch Gutachten 2 lobt diese Veränderung. Außerdem kritisiert Gutachten 1 den beschränkten Altersrange – auch diese Kritik kam bereits in der ersten Begutachtungsrunde auf und wurde vom Fachkollegium vor dem Hintergrund der Machbarkeit eines Längsschnitt-Designs zurückgewiesen. Gutachten 2 sieht nahezu alle Kritikpunkte aus der ersten Begutachtung als hinreichend adressiert an und wiederholt die bereits in der vorherigen Runde zum Ausdruck gebrachte ausgesprochen positive Bewertung des Antrags und des Antragstellers. Lediglich die Frage des Konzepts „Children of Concern" kann die gutachtende Person nicht vollständig überzeugen. Aus Sicht des Fachkollegiums begründet der Antragsteller aber überzeugend, warum er eine präselektierte Risiko-Stichprobe untersucht, was zwar die Generalisierbarkeit der Ergebnisse einschränkt, die Effektstärken des Zusammenhangs zwischen Adversity und Psychopathologie bzw. des Einflusses des LC-NE-Systems auf diesen Zusammenhang aber erhöhen sollte. Auch Gutachten 2 kommt zu einer uneingeschränkten Förderempfehlung. Das Fachkollegium schließt sich der sehr positiven Einschätzung der Gutachtenden an und empfiehlt diesen Antrag zur Förderung."

Allgemeiner Hinweis:

Bitte reichen Sie zukünftige Anträge nur noch in englischer Sprache ein. Angesichts der zunehmenden Vernetzung und Internationalisierung der psychologischen Forschung kann eine unabhängige und sachgerechte Begutachtung bei deutschsprachigen Anträgen nicht immer gewährleistet werden.
